# Establishment of Different Intraoperative Monitoring and Mapping Techniques and Their Impact on Survival, Extent of Resection, and Clinical Outcome in Patients with High-Grade Gliomas—A Series of 631 Patients in 14 Years

**DOI:** 10.3390/cancers16050926

**Published:** 2024-02-25

**Authors:** Franziska Staub-Bartelt, Marian Preetham Suresh Babu, Andrea Szelényi, Marion Rapp, Michael Sabel

**Affiliations:** 1 Department of Neurosurgery, Medical Faculty, Heinrich-Heine University Düsseldorf, Moorenstraße 5, 40225 Düsseldorf, Germany; marion.rapp@med.uni-duesseldorf.de (M.R.); michael.sabel@med.uni-duesseldorf.de (M.S.); 2 Department of Neurosurgery, General Hospital Bamberg, 96049 Bamberg, Germany; smarianpreetham@gmail.com; 3 Department of Neurosurgery, LMU University Hospital, LMU Munich, 80539 München, Germany; andrea.szelenyi@med.uni-muenchen.de

**Keywords:** intraoperative neuromonitoring, brain mapping, supratentorial brain tumor, eloquent brain tumor, surgical approach, infiltrating tumor

## Abstract

**Simple Summary:**

Glioblastoma is the most prevalent intracranial tumor in adults, and simultaneously the most aggressive. Surgical resection constitutes the initial step in the therapeutic approach, and is also highly significant, as studies have demonstrated that overall survival is markedly influenced by the extent of resection or residual tumor volume. Over the past few decades, various techniques for preoperative planning and intraoperative functional monitoring have been introduced to enhance the extent of resection, particularly in the case of functionally eloquent tumors. In this study, we conducted a monocentric investigation into the impact of various intraoperative surgical techniques within the realm of neurophysiological monitoring and mapping, introduced sequentially, on the overall survival of glioblastoma patients. In our cohort of 631 patients, each technique described did not exhibit a significant influence on overall survival.

**Abstract:**

BACKGROUND: The resection of brain tumors can be critical concerning localization, but is a key point in treating gliomas. Intraoperative neuromonitoring (IONM), awake craniotomy, and mapping procedures have been incorporated over the years. Using these intraoperative techniques, the resection of eloquent-area tumors without increasing postoperative morbidity became possible. This study aims to analyze short-term and particularly long-term outcomes in patients diagnosed with high-grade glioma, who underwent surgical resection under various technical intraoperative settings over 14 years. METHODS: A total of 1010 patients with high-grade glioma that underwent resection between 2004 and 2018 under different monitoring or mapping procedures were screened; 631 were considered eligible for further analyses. We analyzed the type of surgery (resection vs. biopsy) and type of IONM or mapping procedures that were performed. Furthermore, the impact on short-term (The National Institute of Health Stroke Scale, NIHSS; Karnofsky Performance Scale, KPS) and long-term (progression-free survival, PFS; overall survival, OS) outcomes was analyzed. Additionally, the localization, extent of resection (EOR), residual tumor volume (RTV), IDH status, and adjuvant therapy were approached. RESULTS: In 481 patients, surgery, and in 150, biopsies were performed. The number of biopsies decreased significantly with the incorporation of awake surgeries with bipolar stimulation, IONM, and/or monopolar mapping (*p* < 0.001). PFS and OS were not significantly influenced by any intraoperative technical setting. EOR and RTV achieved under different operative techniques showed no statistical significance (*p* = 0.404 EOR, *p* = 0.186 RTV). CONCLUSION: Based on the present analysis using data from 14 years and more than 600 patients, we observed that through the implementation of various monitoring and mapping techniques, a significant decrease in biopsies and an increase in the resection of eloquent tumors was achieved. With that, the operability of eloquent tumors without a negative influence on neurological outcomes is suggested by our data. However, a statistical effect of monitoring and mapping procedures on long-term outcomes such as PFS and OS could not be shown.

## 1. Introduction

IDH-wildtype Glioblastoma (GBM) is the most aggressive form of glioma and the most common primary malignant brain tumor, accounting for 16% of primary brain and central nervous system (CNS) neoplasms [1]. The worldwide incidence varies [2], with an average age-adjusted incidence of GBM of 3.2 per 100,000 population in the USA [3]. GBM is primarily diagnosed at older ages, with a median age of diagnosis of 64 years. It is also more prevalent in men than women. GBM is uncommon in children, accounting for approximately only 3% of all brain and CNS tumors reported among patients 0–19 years old [4].

Constant research is performed to identify novel treatment strategies that increase the OS period of patients while ameliorating their quality of life, as the prognosis still is very limited. Despite maximum treatment, the recurrence of GBM following surgical resection is nearly inevitable, and its management is typically not standardized, but rather case-dependent [5,6].

Currently, the standard of care for patients with GBM comprises surgical resection followed by radio and chemotherapy [7,8,9], with emphasis on the importance of the surgical approach. Since GBM is an infiltrating tumor, surgical excision is often challenging. However, maximizing the resection of the tumor plays an important role in the prognosis of the disease [10,11,12]. The extent of resection threshold >80% was shown to be beneficial in primary and recurrent high-grade gliomas [13,14]. Over the years, advances in “supratotal” resection, meaning resection beyond contrast-enhancing tumor margin, have also been commonly discussed in both low-grade and high-grade glioma surgery. In glioblastoma patients, evidence was found that supratotal resection increased survival benefit [11,15,16].

Hence, with the manifesting importance of surgical approaches, more techniques have evolved to push the limits of gross total resection. The introduction of 5-Aminolevulinic acid (5-ALA) as an intraoperative tool for the visualization of the tumor increased progression-free survival and results of tumor resection [17]. However, there is a significant demand for intraoperative techniques that enable the preservation of neurological integrity in the patients as postoperative new neurological deficits are well known to decrease survival benefits [18]. For the control of motor and sensory functionality, intraoperative neuromonitoring (IONM) and monopolar mapping procedures are well-established procedures during brain tumor resection [19,20,21,22]. Monitoring techniques with transcranial electric stimulation of motor-evoked potentials (MEP) as well as monopolar direct cortical stimulation are used for the control of motor function [23,24,25], whereas somatosensory-evoked potential (SSEP) monitors the primary sensory cortex by stimulating peripheral nerves along the somatosensory afferent pathways that lead to the primary somatosensory cortex. The inception of awake craniotomy in glioma surgery additionally enables speech monitoring via bipolar mapping during surgery [26,27].

As we—one by one—have implemented different monitoring and mapping techniques as well as awake surgery in our surgical procedures over the past few years, we aimed to investigate the relevance and impacts of different technical approaches on the survival and outcome of GBM patients. For the present study, we retrospectively screened data from 1010 patients who underwent surgery at our department between 2004 and 2018. In total, 631 patients were included in a detailed study, analyzing the operative techniques that might have increased or decreased the preservation of neurological functionality and whether these intraoperative neurophysiological techniques played a significant role in improving the PFS and OS of patients with GBM or IDH-mutated astrocytoma WHO grade 4.

## 2. Patients and Methods

The present study received approval from the local ethics committee of the University Hospital of Duesseldorf (Study-Nr. 2018-79-RetroDEuA) and was performed as a retrospective patient analysis. All patients enclosed in this study gave written informed consent on data processing within the neurosurgical working group for different scientific issues. The local ethics committee approved the informed consent document and procedure as appropriate for this study.

The authors want to emphasize that the used terms for histopathological diagnosis were adapted to the latest WHO classification published in 2021 [28], even though the observation time ended in 2018. Patients formerly classified as IDH-mutated glioblastoma and IDH-wildtype glioblastoma were included in the study. In the following, the authors will use the term “IDH-mutated astrocytoma WHO grade 4” for all patients in the cohort who were formerly diagnosed with IDH-mutated glioblastoma. IDH-wildtype glioblastoma patients remained the same and are referred to as “GBM”.

The systematic data screening of patients who underwent primary surgery at the Department of Neurosurgery at University Hospital Düsseldorf from 1 January 2004 to 31 December 2018 was performed. Patient recruitment was conducted through “Medico”, the local patient data management system (CompuGroupMedical, CGM Clinical Europe GmbH, Koblenz, Germany). Using the C71 classification code from the International Classification of Diseases (ICD), search criteria were created to generate a list of all cerebral neoplasms. From this list, all patients with a diagnosis of IDH-wildtype glioblastoma and IDH-mutated astrocytoma WHO grade 4 were selected (formerly IDH-mutated glioblastoma), resulting in 1010 patients. The verification of the date of death was performed by either screening the local patient data management system, registering queries, or contacting the patients’ families. This procedure was in line with the obtained local ethics approval. 

Then, we defined further exclusion criteria for the analysis to obtain comparable data:

1. Patients under 18 years at time of diagnosis;
2. Primary surgery at external hospital;
3. Loss of follow-up < 3 months;
4. Incomplete clinical data (NIHSS, KPS, MRI).

**Figure 1:**
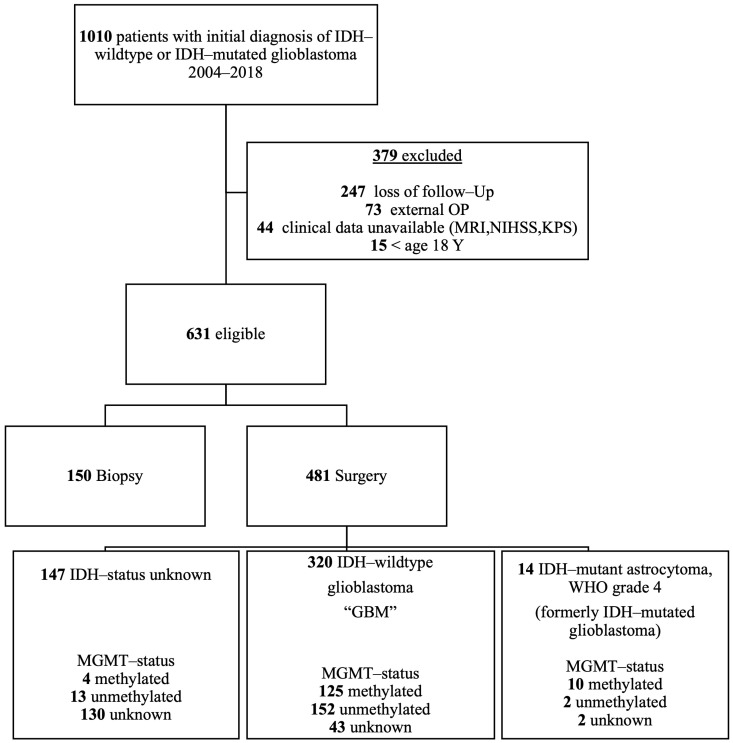
Flowchart of patient selection for analysis with numbers of drop outs at each stage.

After applying the exclusion criteria, a cohort of 631 eligible patients remained (Figure 1).

### 2.1. Surgical Procedure—Resection vs. Biopsy and Categories of Surgical Monitoring and Mapping Approaches

Eligible patients were divided into two major subgroups according to the surgical procedure: “surgical resection” and “biopsy”. The biopsy group was excluded from further statistical analysis as the major focuses of the study were PFS and OS. The comparison of biopsy-only patients to patients who underwent surgery in the context of PFS and OS would have been extremely biased. However, we included patients with biopsies for a description of their distribution from 2004 to 2018 to compare their frequencies in correlation with the implementation of different operative settings over the years. 

Patients from the resection group additionally were analyzed according to the type of technical surgical approach.

The types of intraoperative neurophysiological mapping and monitoring techniques were categorized as follows:

SP-O—Surgical procedure only, with no added monitoring and/or mapping modalities;

AWAKE-bipolar—Awake craniotomy with 60 Hz bipolar stimulation;

IONM-monopolar—Surgery under general anaesthesia under usage of SSEP, MEP, and EEG monitoring, and additionally monopolar mapping;

AWAKE-IONM-mapping—This method incorporated all techniques, such as awake craniotomies with monitoring of SSEP and MEP as well as mapping procedures via bipolar and monopolar stimulation, with a focus on bipolar mapping for speech and language assessment and monopolar mapping for motor control

The determination of the chosen surgical setting was given following the consideration of the lead surgeon(s). During the observation period, the same three surgeons were mainly leading the surgeries (two senior physicians, and one resident who later on also practiced as a senior physician). All of them received specialized training in tumor surgery. Localization of the lesion, evaluation of MRI scans, and neurological status as well as compliance of the patients were evaluated pre-surgery.

### 2.2. PFS and OS

Progression-free survival was determined by an evaluation of RANO criteria for high-grade glioma [29] in the post-operative and follow-up MRIs. The date of MRI was set for the diagnosis of progress if applicable. OS was calculated from the individual death data of the patients. PFS and OS were calculated for the resection cohort regardless of adjuvant treatment, and PFS and OS were compared between the different surgical approaches. In a further subgroup analysis, patients from the resection group that were treated with the STUPP scheme in an adjuvant setting were separately analyzed concerning PFS and OS; here also surgical approach subgroups were compared to each other.

### 2.3. Further Variables of Data Collection

#### 2.3.1. IDH Status and Adjuvant Therapy of Resection Group

For descriptive analysis, patients from the resection group were also divided into three sub-groups based on their IDH statuses. Group one included IDH-mutated patients, now referred to as IDH-mutated astrocytoma WHO grade 4; group two were IDH-wildtype glioblastoma patients, and group three were patients without a known IDH status. Within these subgroups, sub-classification related to MGMT status was performed, resulting in three additional subgroups: MGMT-methylated, MGMT-unmethylated, and MGMT-unknown. Additionally, adjuvant therapy was performed.

#### 2.3.2. Eloquence of Tumor Localization

Although to some extent, the entire brain can be considered eloquent, for this study, the authors defined brain areas as eloquent if they suffered a predictable detectable loss of language, motor, or sensory function when affected by tumor growth or surgical approach. These included the right and left pre- and post-central cortex, the basal ganglia, the calcarine cortex, Wernicke’s area, and areas of language function. The terms eloquent and functional tissue are used interchangeably.

#### 2.3.3. Extent of Resection and Residual Tumor Volume

For the definition of EOR and residual tumor volume (RTV), contrast enhancement in the pre-operative T1-weighted MRI was compared to residual contrast enhancement in the post-operative T1-weighted MRI. Tumor volumetry was performed using either computer software (Brainlab, Elements, Smartbrush, Munich, Germany) or by measuring using the ABC/2 formula [30] if the Brainlab Software was not available. The EOR and RTV were consistently assessed by a single team member within the neuro-oncology team to avoid bias among various evaluators. An EOR, measured as a percentage reduction of tumor volume, of 95% or greater in the post-operative MRI was defined as near total contrast enhancement (CE) resection. Subtotal CE resection was achieved in cases with >80%–95% of CE resection + <5 mL residual tumor volume [31]. Percentage values of the mean, median, and range were calculated for the EOR. RTV was measured as residual contrast enhancement in the post-operative MRI and calculated in milliliters. EOR measured by RTV was categorized, according to Karschnia et al., into class 2 maximal CE resection and class 3 submaximal CE resection, while classes 1 and 4 EOR measured by RTV were not part of the cohort [11]. “Supratotal” resection, meaning the resection of contrast enhancement surrounding tissues with conspicuous findings in the MRI T2 or FLAIR sequence, was not the target of analysis, as this surgical approach was introduced relatively late in terms of the present data collection, and in earlier years of our cohorts’ data, this concept was not standardized.

#### 2.3.4. Neurological Outcome

To evaluate neurological outcomes in patients, we applied the National Institutes of Health Stroke Scale (NIHSS) pre-surgery, post-surgery at time of discharge, and 3 months after surgery. Additionally, we used the Karnofsky Performance Scale (KPS) to assess the functionality of all patients at the above-mentioned time points. NIHSS data were grouped into score values of <5, 5–10, and >10 for the easier illustration of results. The KPS was divided for group analysis into <60%, then segmented in 10-point increments up to 100%. KPS and NIHSS were calculated with median values and their respective interquartile ranges for all included time points.

### 2.4. Statistical Analyses

Descriptive statistics with the mean, and medians with interquartile ranges and standard deviations, were calculated using the statistical function available in Microsoft Excel. Bar and line graphs generated from Excel were used to extrapolate the values graphically. For special statistical analyses, the Statistical Package for the Social Sciences (IBM Corp. Released 2019, IBM SPSS Statistics for Windows, Version 26.0., Armonk, NY, USA, IBM Corp.) was used. A Pearson chi-square test was used to determine whether the frequencies of different intraoperative techniques were statistically significant when compared to others. The one-way ANOVA was used to test the statistical significance of EOR, NIHSS, and KPS in different surgical settings. Although this test allowed us to determine the statistical significance of the means of the independent groups, repeated-measure ANOVA was required to test the relationships of the means between the independent groups, as repeated-measure ANOVA is a mode of analysis of dependencies. To analyze the distribution of survival, a Kaplan–Meier survival curve was used to determine the distribution of PFS and OS. Statistical significance was set at *p* < 0.05.

## 3. Results

Out of 1010 screened patients, 631 were considered eligible for the study. Of these, 150 were biopsied, and the remaining 481 patients underwent primary surgical resection. Up to the last individual observation time, 165 patients underwent recurrent surgery, 60 patients had a second recurrent surgery, 19 patients had a third surgery, 4 patients received a fourth surgery and 1 patient had five surgeries. The results are presented for the primary resection only. In total, 303 of the 481 patients were male (63%) and 178 were female (37%). The median age at diagnosis for the resection group was 61 years (IQR 52.8–70.0); 94% of the patients underwent surgery using 5-ALA fluorescence. For a summarized description of the resection cohort and its surgical subgroups, please refer to Table 1. 

### 3.1. Surgical Characteristics and their Changes from 2004 to 2018

Out of 631 patients, 150 underwent biopsy only (24%) and 481 (76%) underwent primary resection. In the resection group, SP-O was performed in the majority of patients (n = 160, 33%) compared to surgery with intraoperative monitoring and monopolar mapping (IONM-monopolar), which was seen in 145 out of 481 patients (30%). Surgery under usage of all available methods (AWAKE-IONM-mapping) was seen in 22% of the patients (n = 105). AWAKE-bipolar surgery was performed in 71 patients (15%).

**Figure 2:**
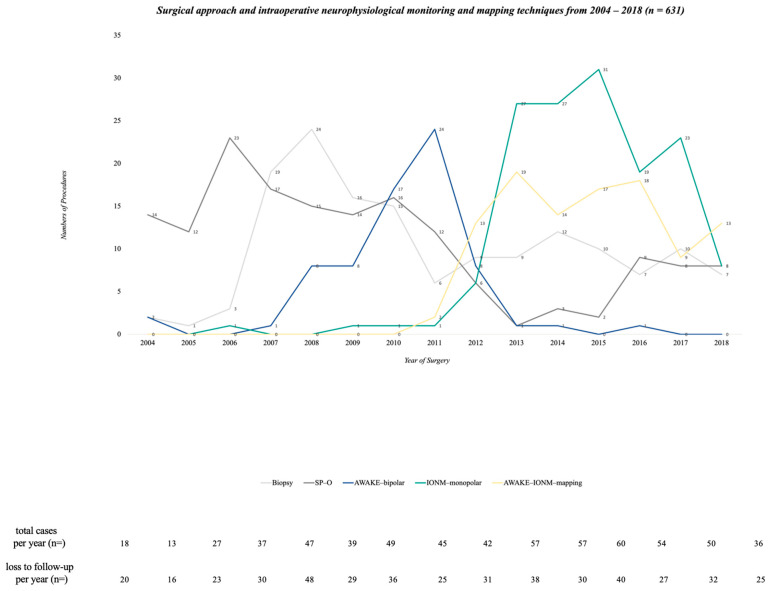
Line graph of patients enclosed in the analysis (n = 631) divided by technical settings from 2004 to 2018. Extra information is given in the footnotes concerning the total number of cases and the cases that were lost to follow-up per year.

A year-wise extrapolation of the technical settings was performed for a better understanding of the changes in surgical settings over time. Towards the end of 2011, the number of SP-O decreased. In 2007, awake surgeries were implemented with 60 Hz stimulation, and their numbers steadily increased until 2011. IONM-monopolar and the combination of AWAKE-IONM-mapping were implemented in 2010, and over time, this became the institutional standard of surgical care for patients. As IONM-monopolar increased, SP-O decreased considerably. As seen in the graphical representation, the period from 2010 to 2012 marked a pivotal change in defining the standard of care for surgery for patients with GBM (Figure 2). The chi-square test showed that the performance of biopsies decreased significantly after 2010, along with a statistically significant increase in AWAKE-bipolar, IONM-monopolar and AWAKE-IONM-mapping surgical settings after 2010 (*p* < 0.001).

### 3.2. PFS and OS in the Surgical Cohort

**Figure 3:**
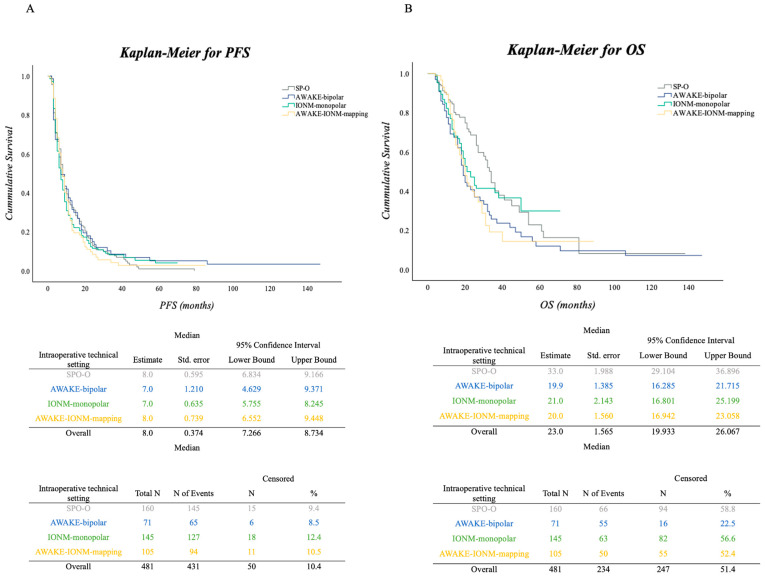

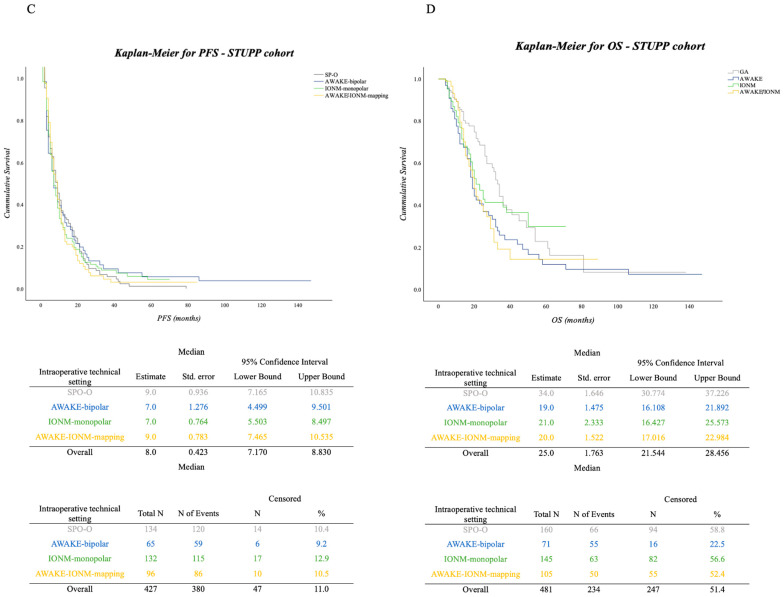
(**A**,**B**): Kaplan–Meier curves for PFS (**A**) and OS (**B**) for the cohort with median OS for all surgical technique subgroups, along with number of events and number censored as well as 95% CI in the footnotes. There was no significant difference concerning intraoperative mapping or monitoring techniques in PFS (*p* = 0.749). Kaplan–Meier curve for OS (**B**) for the cohort with median OS for all subgroups, number of events and number censored as well as 95% CI in the footnotes. There was an increased OS in patients who underwent surgery via SP-O technique compared to the other surgical techniques (*p* = 0.034). (**C**,**D**): Kaplan–Meier curves for PFS (**C**) and OS (**D**) for the STUPP-subcohort with median OS for all surgical technique subgroups, number of events and number censored as well as 95% CI in the footnotes. There was an increased OS in patients who underwent surgery via the SP-O technique compared to the other surgical techniques (*p* = 0.034).

To calculate the PFS and OS, Kaplan–Meier survival curves were plotted for the different operative settings. The median follow-up of the cohort was 14 months. The median PFS was 8 months (IQR 4–15). There was no significant difference in the PFS under the various surgical settings in the log-rank (Mantel-Cox) test (*p* = 0.749). The median OS for the resection cohort was 23 months (IQR 14–50). There was a significantly increased OS associated with SP-O surgical approach compared to the other surgical approaches/techniques (*p* = 0.034). Figure 3A,B illustrate Kaplan–Meier curves for PFS and OS for the entire cohort, with median survival, number of events and number censored for the cohort and all surgical subgroups.

#### Impact of Adjuvant Therapy in Surgical Approaches concerning PFS and OS

From the surgical cohort, 427 patients underwent adjuvant treatment. Out of 320 IDH-wildtype patients, 296 underwent at least one circle of treatment from the STUPP-protocol, and out of the 14 patients with mutated IDH statuses, 12 were treated with at least one circle of STUPP-scheme. More detailed findings on adjuvant therapies for all patients according to their surgical approaches are made available in Table 2.

We performed a subgroup analysis for the STUPP cohort of 427 patients to reveal a possible influence of adjuvant therapy on PFS and OS. There was no significant increase in PFS for any of the surgical approach subgroups (median PFS for all subgroups in months = 8; SP-O—9, AWAKE-bipolar—7, IONM-monopolar—7, AWAKE-IONM-mapping—9; *p* = 0.923). Regarding OS, we found statistically significantly increased OS in patients associated with the SP-O group for patients receiving adjuvant STUPP compared to the other surgical approaches (median OS for all subgroups in months = 25; SP-O—34, AWAKE-bipolar—19, IONM-monopolar—21, AWAKE-IONM-mapping—20; *p* = 0.034). The details of this subgroup analysis can be found in Figure 3C,D.

### 3.3. Tumor Localization and Eloquence

A total of 405 patients had eloquently located tumors. In order to compare the functional locations between different surgical methods, we chose to extrapolate the distribution of eloquently located tumors by years comprising the whole screening period. Statistical analysis showed that since the implementation of the AWAKE-bipolar and/or IOMN-monopolar settings after 2010, the total number of surgeries of eloquence increased significantly from 75 procedures between 2004 and 2009 to 330 procedures between 2010 and 2018 (*p* < 0.001). Furthermore, the proportion of surgeries comprising resection of functional tissue increased in relation to the total number of operations over the observed period (2004–2009, 116 procedures in total, 64.7% of those being eloquent, 35.3% not eloquent; 2010–2018, 365 procedures in total, of those 90.4% being eloquent and 9.6% not eloquent). The SP-O group was the only subgroup with non-functional tumors. All other groups only included lesions located in functional areas. A summary of location and eloquence is illustrated in Table 1. Concerning the dependence of monitoring or mapping procedures, we analyzed all procedures performed after 2010, when all surgical techniques were available at our department. In line with a previous publication from another cohort that was treated at our department [22], we found that, consistent with the widespread use of mapping and monitoring, 82% of left hemisphere lesions were operated in an awake surgery setting, while in 64%, mapping procedures were performed. Intraoperative neuromonitoring (IONM) using monopolar stimulation was performed, manly for right hemispheric lesions (96%), particularly in frontoparietal and multilocular cases. Table 1 presents a summary of tumor location and eloquence by years and technical setting.

### 3.4. Extent of Resection and RTV

In total, 479 patients received pre- and post-operative MRI to assess the EOR and RTV; 2 patients in the SP-O group did not receive post-op MRI. Mean tumor volume in the preoperative MRI was 35.2 mL ((SD ± 31.8), range 0.3–226.8 mL) for the cohort. Preoperative tumor volume did not differ significantly between the surgical subgroups (*p* = 0.169).

**Figure 4:**
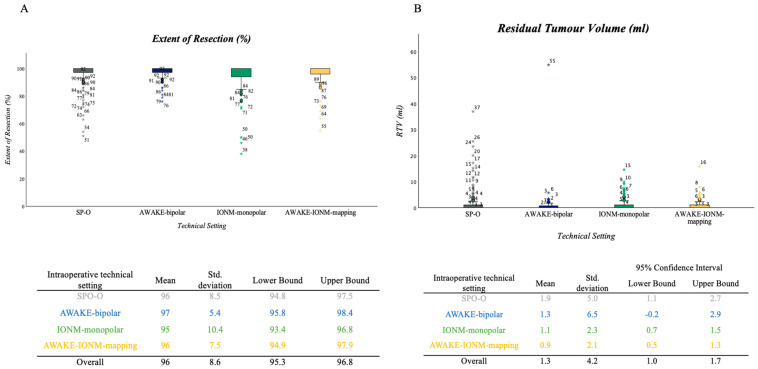
(**A**,**B**) Boxplots. (**A**) EOR (%) compared within the different surgical technique subgroups, showing intraoperative neurophysiological techniques of all cohort patients, and with one-way ANOVA revealing no statistically significant difference (*p* = 0.404, mean EOR in SP-O group was 96%, in AWAKE-bipolar subgroup it was 97%, in the IONM-monopolar cohort it was 95% and in the IONM-AWAKE-mapping subgroup it was 96%). (**B**) RTV (mL) compared with different intraoperative neurophysiological techniques for all cohort patients, and with one-way ANOVA revealing no statistically significant difference (*p* = 0.188). For illustrative purposes, the representation of two statistical outliers has been omitted. The values were group SP-O n = 1 with 37.00 mL residual volume and group AWAKE-bipolar n = 1 with 54.91 mL residual volume.

The mean EOR calculated as a percentage reduction of tumor volume was 96% ((±9 SD) for the entire cohort, range 38–100%); 373 patients (76%) had near-total CE resection, while the remaining 108 (22%) had subtotal CE resection. Of the patients who were operated on via SP-O, 129 showed near-total CE resection and 29 showed subtotal CE resection. In the AWAKE-bipolar setting, 56 patients showed near-total CE resection and 15 subtotal CE resection, and in the IONM-monopolar group, 108 patients had near-total CE resection, and 37 subtotal CE resection. Finally, in those operated on via the AWAKE-IONM-mapping setting, 82 had near-total CE resection and 23 had a subtotal CE resection. The EOR (%) did not show a significant difference when analyzed separately for the years before (2004–2009) and after (2010–2018) the introduction of all IONM and mapping techniques (mean EOR before 95%, mean EOR after 96%). In line with that, no statistical significance (*p* = 0.404) was found regarding the EOR and different surgical settings for the whole period (Figure 4A).

The extent of resection was also assessed as RTV in the postoperative MRI. The mean RTV in the cohort was 1.38 mL (±4.16 SD). The mean RTV measured in the postoperative MRI was the lowest in the AWAKE-IONM-mapping group (0.9 mL (±4.2)) and highest in the SP-O group (1.93 mL (5.0)). No statistical significance (*p* = 0.186) was found regarding the RTV and different surgical settings for the whole period; according to the mean values, all subgroups reached class 3 A (subtotal) resection (Figure 4B, mean RTV for SP-O = 1.93 mL, AWAKE-bipolar = 1.35 mL, IONM-monopolar = 1.1 mL and AWAKE-IONM-mapping = 0.9 mL). Of the patients who were operated on via SP-O, 118 showed class 2 B resection (near-total CE resection), 22 showed class 3 A resection and 18 showed class 3 B resection. In the AWAKE-bipolar setting, 57 patients showed class 2 B resection, and 12 showed 3 A, while 2 patients showed 3 B resection. In the IONM-monopolar group, 106 patients showed class 2 B, and 27 showed class 3 A resection, while 12 patients showed class 3 B resection. Finally, in those operated on via the AWAKE-IONM-mapping setting, 78 had class 2 B resection and 23 had class 3 A resection, while 4 patients showed class 3 B resection.

The RTV also did not show a significant difference when analyzed separately for the years before (2004–2009) and after (2010–2018) the introduction of all IONM and mapping techniques.

### 3.5. Neurological Outcome in Resection Group: NIHSS and KPS

#### 3.5.1. NIHSS

**Figure 5:**
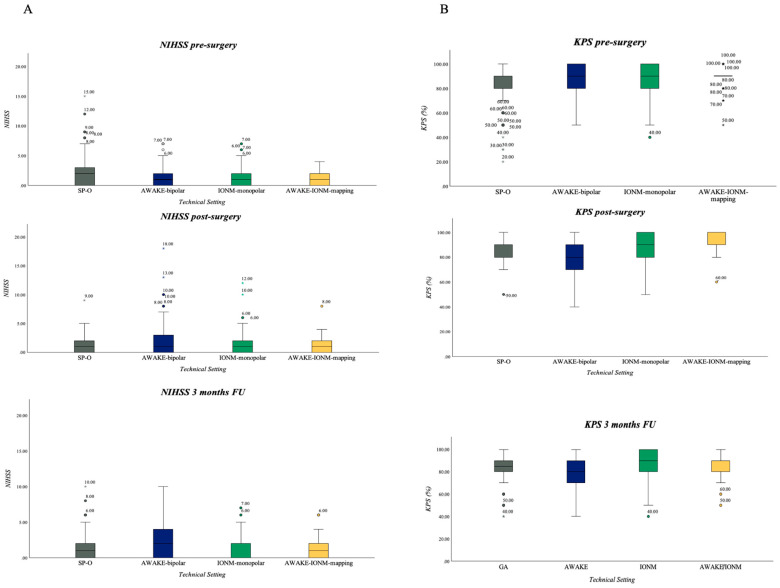
(**A**) Boxplot NIHSS: NIHSS pre- and post-surgery as well as at three-month follow-up for all patients. The median NIHSS scores for the surgical subgroups at different time points were (pre-surgery/post-surgery and at three-months follow-up): SP-O 2/1/1, AWAKE-bipolar 1/1/2, IONM-monopolar 1/1/0, AWAKE-IONM-mapping 1/1/1. (**B**) Boxplot, KPS pre- and post-surgery as well as at three-months follow-up for all patients. The median KPS scores for the surgical subgroups for different time points were (pre-surgery/post-surgery and at three-months follow-up): SP-O 80/80/85, AWAKE-bipolar 90/80/80, IONM-monopolar 90/90/90, AWAKE-IONM-mapping 90/90/90.

Median NIHSS for the entire cohort pre-surgery was 1 (IQR 0–2) and remained the same at all other analyzed time points (Figure 5A). When comparing the subgroups of individual monitoring or mapping techniques, patients in the SP-O group served as the baseline without any monitoring, and were compared to other groups with monitoring and/or mapping techniques. In a post-hoc analysis, it was observed that at the time of postoperative assessment, patients in the “AWAKE-bipolar” group had a significantly higher NIHSS compared to non-monitored patients in the SP-O group (*p* = 0.005). This effect was also evident at the three-month follow-up (*p* = 0.044). In the intergroup comparison, when only the monitoring/mapping groups were considered, the AWAKE-bipolar group also exhibited a significantly higher NIHSS postoperatively and at the three-month follow-up.

#### 3.5.2. KPS

Median KPS pre-surgery for the whole cohort was 90 (IQR 80–100) and remained the same at post-surgical assessment and three-month follow-up. Post-operative analysis showed that the patients operated on under SP-O, IONM-monopolar and AWAKE-IONM-mapping settings experienced no deterioration in terms of their KPS score. However, a significant decrease in the KPS score was observed in patients who were operated on under the AWAKE-bipolar setting, while the KPS score for those who were operated on via SP-O increased (Figure 5B, *p* < 0.001).

The three-month follow-up revealed significantly higher KPS values for the IONM-monopolar (*p* = 0.004) and AWAKE-IONM-mapping (*p* = 0.021) groups compared to those under the SP-O surgical setting, and again, the AWAKE-bipolar group showed a significantly lower KPS score compared to SP-O, as calculated using the Bonferroni method (*p* = 0.012). 

## 4. Discussion

The standards for the surgical treatment of GBM have evolved considerably. In our study, we found significant changes in the intraoperative monitoring techniques used since 2004. We observed three main phases: (1) no monitoring until 2007, (2) the introduction of awake craniotomies with bipolar stimulation as the only monitoring/mapping technique in 2008 and (3) the introduction of IONM and mapping as a combined procedure in 2010. From there on, whenever required, all techniques were used as combined techniques.

In the present study, which spanned 2004–2018, we aimed to quantify the influence of elaborate intraoperative monitoring and mapping methods on the immediate effects measured in neurological outcomes and the long-term prognosis of patients with eloquently located GBM.

### Implementation of Different Surgical Monitoring and Mapping Techniques and Their Impacts on Short-Time and Long-Time Outcomes

In our department, awake surgery with bipolar mapping was the first technique used for the functional monitoring of patients with eloquent tumors, introduced to preserve functionality in patients with eloquent tumors. Awake craniotomies with 60 Hz bipolar stimulation as one technique for eloquent GBM resection provide functional control on the cortical and subcortical levels of a wide array of neurological functions, particularly language functions. Thus, the importance of awake craniotomies has been recognized in the surgical management of eloquently located gliomas in the past, and the implications of this technique for eloquently located GBM have been discussed widely in the literature. In 2008, Sanai et al. reported data on 250 patients who underwent resection under intraoperative language mapping. Further, they reported a worsening of language function in 8.4% of the patients during the postoperative phase. However, after 6 months, only 1.6% of the patients were reported with permanent worsening [32]. Recently, Gerritsen et al. published data from a multicenter study comprising 536 GBM patients with eloquently located lesions; 134 of the patients underwent awake surgery. Overall, patients in the awake subgroup compared to the non-awake group had significantly fewer neurological deficits at three and six months [33]. Comparable results were obtained in a meta-analysis published by Sattari, revealing a significantly greater EOR, longer PFS and OS, as well as higher KPS scores at three months in 2032 eloquent gliomas [34]. In our cohort, we found a median PFS of 8 months. PFS as calculated by Kaplan–Meier curve showed no significant superiority of the technical settings that were used. For OS, a median survival of 14 months was observed for the entire cohort, and here, the Kaplan–Meier curve revealed significant results in the SP-O group. However, the authors want to interpret these results judicially. First and foremost, the SP-O group was the largest, with 160 patients compared to the other subgroups. Several cases might have biased outcomes regarding PFS and OS. Furthermore, SP-O was mostly implemented in patients before 2010, when the significance of IDH-status on the prognosis of GBM was still under investigation. The patients operated on via SP-O in our cohort comprised mostly unknown IDH statuses. Amongst these patients, statistical outliers had an OS of more than the expected normal average life expectancy. The authors can only speculate that a majority of patients in this cohort may have an IDH-mutated status with a known beneficial overall survival (OS), although this cannot be conclusively demonstrated. It should also be noted that the groups were highly imbalanced (14 IDH-mutated vs. 320 IDH-wildtype patients), allowing for trends but not statistically robust conclusions.

The AWAKE-bipolar group in our study exhibited a significantly increased NIHSS and reduced KPS in the postoperative phase compared to the other groups. To further evaluate this phenomenon, we examined factors that could have had some influence on these unexpected results. The eloquence of the operated lesions was not a contributing factor. However, upon investigating the locations, it was found that a quarter of the lesions were located in the parietal lobe. Since, in comparison to the AWAKE-bipolar group, the AWAKE-IONM-mapping group that implemented additional IONM and both mapping techniques did not show a higher postoperative NIHSS, the possible absence of monopolar mapping when addressing motor eloquent areas in the parietal lobe may explain this outcome. However, as the nature of the deficit—motor or language—was not able to be obtained from the raw data, and therefore not further evaluated, the authors can only make conjectures in this regard. 

As there is a distinctive dependence on the patient’s compliance, not all patients can undergo awake surgery. If patients are identified as not suitable for awake craniotomy or in cases where the control of motor function is prioritized over language testing due to localization or clinical findings, IONM and monopolar mapping are profoundly effective techniques for monitoring somatosensory and motor functions in the asleep setting, providing the active monitoring (monopolar stimulation) of cortical or subcortical motor functions.

Combined monitoring/mapping approaches for surgical resection were implemented in our department, starting at the end of 2009. When using IONM with monopolar mapping only or awake surgery combined with those techniques, we did not see any significant increases in NIHSS or decreases in KPS as parameters for new neurological deficits, which is in line with multiple studies reporting save resection under usage of these techniques [20,35,36,37]. After the implementation of these combined techniques, the ratio of biopsies compared to surgeries decreased significantly, and the incorporation of IONM-monopolar and AWAKE-IONM-mapping procedures drastically increased in our cohort. The surgical excision of eloquently located tumors also significantly increased from there on. Nevertheless, as reported in our data, those techniques did not have any influence on PFS or OS in our cohort. Our findings are in line with a study by Fukui et al., which focussed on the impact of awake craniotomy and mapping on OS in a cohort of 335 patients and found no significant results for OS when patients were operated on in the awake setting compared to under general anaesthesia [38]. Additionally, Pan et al. found no statistically significant differences between “IONM” and “no-IONM” groups concerning PFS and OS [39].

Lastly, in a side analysis, we aimed to access possible significant differences in EOR and RTV within technical settings to determine a possible influence on PFS and OS in our cohort, as EOR and RTV are known to be significant factors for prolonged PFS and OS. Maximizing EOR is a well-established concept applied to prolong overall survival (OS). Many studies have proven the significant influence of this approach in the past, e.g., Sanai et al. [13,40] and Lacroix et al. [41]. In a recent study, the RANO Resect Group demonstrated that resections beyond the contrast-enhancing part even more notably impact OS [11]. In our cohort, a mean EOR measured as a percentage reduction of 96% was observed throughout the entire period, regardless of the technical setting employed. The average EOR before the incorporation of IONM or mapping techniques or awake surgery was 95%; after that, we saw an average of 96%. Furthermore, we evaluated the residual tumor volume, which did also not differ significantly between technical settings; however, the lowest RTV was seen in the AWAKE-IONM-mapping group in which all IONM and mapping techniques were incorporated. With the implementation of IONM and or mapping techniques, the authors would have expected more different results favouring IONM and or mapping techniques regarding resection results. However, in our cohort, no significant difference was observed compared to the period without the use of IONM or mapping techniques. An important factor to consider here is that with the introduction of these techniques, the eloquence of tumors significantly increased. Comparing EOR or RTV in patients without eloquently located tumors is challenging when compared to those with motor or language-relevant tumors. For non-eloquently located tumors, resection can be guided radiologically/anatomically. But, with eloquent tumors, the aim is to avoid functional deficits, necessitating the acceptance of functional resection boundaries. This could have contributed to the very similar outcomes between the SP-O and various AWAKE and IONM-mapping groups.

Overall, the equally distributed EOR and RTV may have resulted in a non-significant impact of the monitoring/mapping techniques on PFS and OS, as it is known that the extent of resection highly impacts survival in high-grade patients. Since there were no differences in the cohort regarding the extent of resection, the influence on OS of the respective monitoring/mapping techniques could probably not be shown.

## 5. Limitations

This study has certain limitations that the authors want to point out. The retrospective study is based on data spanning 14 years, with some data acquisition dating back significantly into the past. Meanwhile, changes in documentation forms and standards have occurred, posing challenges to comprehensive data collection and meaningful analysis. For example, the chosen intraoperative technical setting was naturally dependent on the standard technology available at the time of data collection. This factor influenced patient allocation to different technical groups until the introduction of all mapping and monitoring techniques after 2010. Notably, out of over 1000 screened patients, only just over 600 were included in the study. Additionally, since the first patient’s surgery, intraoperative techniques, excluding the monitoring and mapping approaches under investigation in this study, have evolved. The introduction and routine use of fluorescence techniques like 5-ALA, or increasingly advanced microscopes, intraoperative imaging, and preoperative planning over the years, have shifted the boundaries of surgeries, likely introducing a certain bias into the data. Moreover, the group sizes of the surgical approaches have varied significantly, making comparisons challenging in some instances. A prospective setting of data acquisition and analysis would have certainly resulted in significantly less data loss, thereby allowing for a substantial increase in both the total number of patients and the data available for analysis. Additionally, the matching of patients would have been simplified, helping to minimize the numbers and impacts of confounders.

## 6. Conclusions

Several publications have advocated for the utilization of intraoperative neuromonitoring (IONM), mapping, and awake craniotomies in the resection of glioblastoma in smaller patient cohorts. However, to our knowledge, there has been no analysis of comparable cohort sizes in the current study that examines the influence of different intraoperative monitoring and mapping techniques on PFS and OS. We observed a significant decrease in the number of biopsies following the incorporation of awake surgeries, intraoperative IONM, and mapping. This decrease suggests a considerable increase in the operability of eloquent tumors since the adoption of awake craniotomies and IONM or mapping techniques.

Therefore, employing these techniques facilitates the safe resection of eloquent GBM with acceptable post-operative morbidity. Nevertheless, our cohort did not demonstrate a significant impact of one of these various surgical monitoring and mapping techniques alone on PFS and OS. Although specific individual techniques did not show a significant impact, our study highlights the importance of intraoperative monitoring and mapping techniques in enhancing overall resectability in GBM, which now provides an opportunity for surgery to some patients who previously would not have been candidates for surgery.

## Figures and Tables

**Table 1:** Summary of resection cohorts’ descriptive data (surgical subgroups and overall cohort).

Characteristic		SP–O	AWAKE–Bipolar	IONM–Monopolar	AWAKE–IONM–Mapping	Overall Resection Cohort
		n = 160	n = 71	n = 145	n = 105	n = 481
Sex						
	male	97	50	88	68	303 (63%)
	female	63	21	57	37	178 (37%)
Age at diagnosis, y						
(*p* = 0.281)	Median (IQR)	62.1 (17.4)	60 (17.7)	60 (19.0)	64 (15.0)	61.0 (52.8-70)
	range	23.0–89	24–80	24–85	29–86	23–89
ALA						
(*p* = 0.281)	Administered	142	70	136	104	452 (94%)
	Not administered	18	1	9	1	29 (6%)
IDH-Status						
(*p* < 0.001)	Wildtype	50	36	134	100	320 (66.5%)
	Mutant	3	2	6	3	14 (2.9%)
	Unknown	107	33	5	2	147 (30.6%)
MGMT-Status						
(*p* < 0.001)	MGMT +	22	7	60	50	139 (28.9%)
	MGMT -	30	15	75	46	166 (34.5%
	Unknown	107	49	10	9	176 (36.6%
KPS (mean)						
	preoperative	82	87	89	90	86.78 (±12.34)
	postoperative	85	77 (*p* < 0.001)	90	91	86.78 (±11.08)
	3 months	82	77 (*p* = 0.012)	88 (*p* = 0.004)	88 (*p* = 0.021)	84.57 (±13.22)
NIHSS (mean)						
	preoperative	1.9	1.6	1.4	1	1.4
	postoperative	1.6	2.5 (*p* = 0.005)	1.2	1	1.3
	3 months	1.6	2.3 (*p* = 0.044)	1.3	1.4	1.6
Pre- OP Tumour volume (mL)						
(*p* = 0.288)	Mean (SD)	37.99 (±28.14)	36.83 (±43.32)	35.97 (±33.21)	29.09 (±24.64)	35.2 (±31.8)
Extent of resection, % by volume						
(*p* = 4.04)	Mean	96%	97%	95%	96%	96.1 (8.6%)
	Median	100%	100%	100%	100%	
	Range	51–100%	76–100%	38–100%	55–100%	38.2–226
Residual volume (mL)						
(*p* = 0.186)	Mean (±SD)	1.95 mL (±5.1)	1.36 mL (±6.58)	1.11 mL (±2.26)	0.91 mL (±2.06)	1.37 (±4.16)
	Range	0–37 mL	0–54.91 mL	0–14.64 mL	0–15.83 mL	0–54.91
Tumour location by hemisphere						
(*p* = 0.001)	Bilateral	4	0	4	1	15 (3.1%)
	Left	60	42	40	82	223 (46.4%)
	Right	96	29	96	21	243 (50.5%)
Eloquence						
(*p* < 0.001)	Eloquent	84	71	145	105	405 (84.2%)
	Not eloquent	76	0	0	0	76 (15.8%)
Before 2010						
	Eloquent					75 (64.7%)
	Not eloquent					41 (35.3%)
After 2010						
	Eloquent					330 (90.4%)
	Not eloquent					35 (9.6%)

**Table 2:** Details on adjuvant therapy, if available, sub-grouped according to the surgical approaches/intraoperative techniques, TMZ = Temodal. Information regarding the initiation of adjuvant therapy in the form of combined radiochemotherapy was derived from the analyzed data from 427 patients. Details about the extent of this therapy were extracted from the data for a total of 321 patients. Subsequently, these patients were categorized into subgroups based on the surgical technique employed.

Adjuvant Therapy (n = 427)	SP–O	AWAKE–BIPOLAR	IONM–Monopolar	AWAKE–IONM-Mapping
Total Number	n = 134	n = 65	n = 132	n = 96
Details Concerning Adjuvant Therapy Available from	n = 75	n = 52	n = 113	n = 81
Stupp complete (60 Gy radiation + concomitant TMZ + adjuvant 6 cycles Temodal	22	14	16	14
Radiation + concomitant Chemotherapy applied, adjuvant TMZ cancelled after initiation	23	15	52	35
Radiation + concomitant Chemotherapy finalised, adjuvant TMZ not initiated	18	22	33	25
Radiation + concomitant Chemotherapy cancelled, adjuvant TMZ not initiated	3	0	2	2
Radiation + concomitant Chemotherapy cancelled, adjuvant TMZ finalised (at least 6 cycles)	1	0	2	0
Herrlinger scheme	8	1	7	5
Radiation only	0	0	1	0

## Data Availability

Data are available on request due to privacy restrictions.
